# Challenges in severe asthma: Do we need new drugs or new biomarkers?

**DOI:** 10.3389/fmed.2022.921967

**Published:** 2022-09-27

**Authors:** Adil Adatia, Harissios Vliagoftis

**Affiliations:** ^1^Department of Medicine, University of Alberta, Edmonton, AB, Canada; ^2^Alberta Respiratory Centre, University of Alberta, Edmonton, AB, Canada

**Keywords:** severe asthma, biomarkers, biologics, eosinophils, anti-IL 5

## Abstract

Severe asthma is a complex, heterogenous airway condition. There have been significant advances in severe asthma management in the past decade using monoclonal antibody therapies that target the inflammatory component of the disease. Patient selection has been paramount for the success of these biologicals, leading to significant interest in biomarkers to guide treatment. Some severe asthmatics remain suboptimally controlled despite trials of biologicals and many of these patients still require chronic systemic corticosteroids. New therapeutics are currently in development to address this unmet need. However, whether these patients could be better treated by using novel biomarkers that inform selection among currently available biologics, and that objectively measure disease control is unclear. In this review, we examine the currently used biomarkers that guide severe asthma management and emerging biomarkers that may improve asthma therapy in the future.

## Introduction

Asthma is a complex respiratory disease characterized by airway inflammation, bronchial hyperresponsiveness, and variable airflow limitation (1). Globally it is among the most common chronic diseases affecting 300 million people worldwide (2), up to 10% of whom have severe disease. Severe asthma is typically defined the need for high dose inhaled corticosteroid therapy and an additional controller medication to prevent loss of disease control, or poor disease control despite these medications (3).

There are two widely recognized asthma endotypes based on the presence or absence of type 2 (T2) airway inflammation (4). In T2-high asthma, clinical symptoms result from inflammation driven by the cytokines interleukin-4 (IL4), IL5, and IL13, as well as alarmins [thymic stromal lymphopoietin (TSLP), IL25, IL33] and Immunoglobulin E (IgE). Clinically, patients with T2-high asthma are recognized using biomarkers that reflect the activity of T2 cytokines such as fractional exhaled nitric oxide and airway eosinophilia (5). T2-low asthma remains poorly defined and generally encompasses patients in whom such markers are not found.

Severe asthma care has been revolutionized by biologics targeting T2 inflammation. There are currently six biologics approved by the Federal Drug Administration for the treatment of severe asthma. Three biologics inhibit eosinophils by blocking IL5 signaling. Mepolizumab and reslizumab target IL5 itself and benralizumab targets the IL5 receptor and depletes eosinophils by antibody-dependent cell-mediated cytotoxicity. Dupilumab interrupts IL4 and IL13 signaling by targeting IL4R alpha, the alpha subunit common to both the IL4 and IL13 receptors. Omalizumab targets free IgE and downregulates expression of the high-affinity IgE receptor FcεR1, and tezepelumab targets TSLP, which is upstream of the other molecules in the T2 airway inflammation cascade.

Precision medicine can be viewed as a process by which comprehensive phenotyping of patients is used to develop novel patient stratification systems, diagnostic and prognostic models, and, ultimately, predict treatment responses (6). Identification of biomarkers, any physiologic parameter that provides an indication of normal biological function, presence of disease, or response to therapy, is a key component of the precision medicine. The ideal biomarker for severe asthma would be non-invasive, reproducible, respond to changes in clinical status (e.g., exacerbations and treatment changes), and inform treatment choice. We have some effective approaches to phenotype patients and are beginning to understanding asthma endotypes, but an important clinical question that perplexes practitioners remains unanswered to a great extent, namely how can we choose the right medication, especially when it comes to biologics, for the right subject with severe asthma to maximize the chances of a clinical response. Phenotyping or endotyping these patients using clinical features and biomarkers is not yet an easy proposition and, in most cases, it is difficult to achieve, even in specialized centers.

The ideal of precision medicine guides many of us when we discuss our current approach to severe asthma therapy, but are we there yet? Do we actually have a way to choose the right therapeutic option for the right patient?

## Current biomarkers

In this section we will discuss the state of current severe asthma biomarkers that inform the use of biologics. The utility of commonly employed laboratory markers in the prediction of response to presently available asthma biologics that are discussed below are summarized in Table 1.

**Table 1:** Summary of the evidence for the utility of currently used biomarkers to predict the efficacy of biologicals in asthma.

	**Blood Eos**	**Sputum Eos**	**Total IgE**	**FeNO**
Omalizumab	?	✓*	—	?
Anti-IL5 (R)	✓*	✓	—	—
Dupilumab	✓*	?	—	✓
Tezepelumab	✓*	?	—	✓

✓, Biomarker useful to predict efficacy; ✓^*^, Biomarker may be helpful but there are significant limitations (see text for details); ?, Insufficient or conflicting data; —, Biomarker does not appear to be useful to predict efficacy.

### Sputum cytometry

Induced sputum is the reference standard for assessing airway inflammation in asthmatics. Sputum is obtained after patients inhale nebulized hypertonic saline, and the expectorate is centrifuged and stained. The granulocytes are then quantified to determine the type of airway inflammation. Based on the percentage of eosinophils and neutrophils, the nature of airway inflammation can be classified as eosinophilic (eosinophils >3%), neutrophilic (neutrophils >64% and total cell count >9.7 × 10^6^ cells/g), mixed granulocytic (both eosinophilic and neutrophilic), and paucigranulocytic (neither eosinophilic nor neutrophilic) (7). Sputum cell counts are reproducible (8) and reflect changes in therapy and disease control, with a minimum clinically important difference in eosinophils of just ~4 percentage points (9). Serial measurements can therefore be highly informative and may be necessary in some patients. For example, systemic corticosteroids and infections may mask airway eosinophilia, and these patients could be misclassified as non-eosinophilic if repeated measurements are not obtained. Similarly, though absent at baseline, sputum eosinophilia may emerge during exacerbations (10).

Clinical trials have clearly established that anti-IL5 therapy is effective if the asthma phenotype is driven by luminal eosinophils and that sputum cytometry can reliably identify this subpopulation. Initial studies of anti-IL5 therapy in broad asthma populations failed to show benefit (11–13), but a subsequent pilot study showed that if patients with sputum eosinophilia are specifically selected, mepolizumab has a significant prednisone-sparing effect (14, 15). Subsequent clinical trials have confirmed the strong predictive value of sputum eosinophilia for anti-IL5 responsiveness (16, 17). Some patients, particularly those dependent on systemic corticosteroids, may continue to have exacerbations driven by eosinophilic bronchitis despite mepolizumab or reslizumab therapy (18). Sputum cytometry appears to be the only effective means to identify this phenomenon, and without this tool clinicians may erroneously attribute the exacerbations to other factors such as viral infections. Benralizumab, owing to its depletion of eosinophils in addition to the blockade of IL5 signaling, may be useful for such patients.

For omalizumab, the severity of a patient's asthma appears to impact the interpretation of sputum eosinophilia as a predictor for treatment response. In mild-moderate asthma, omalizumab effectively ameliorated airway inflammation in patients with luminal eosinophils (19). However, an inadequate response to omalizumab was seen in asthmatics with persistent sputum eosinophilia who remain uncontrolled despite high-dose inhaled corticosteroids with or without systemic corticosteroids (20). There are also no randomized controlled trials (RCTs) showing a systemic steroid-sparing effect of omalizumab. Thus, in the severe population, the presence of persistent sputum eosinophilia may indicate that omalizumab is not the optimal choice of biologic.

There are little data regarding the predictive capacity of sputum eosinophilia for the efficacy of dupilumab. The phase 3 trials did not obtain sputum measurements (21, 22) except for the LIBERTY VENTURE trial, which did not report outcome measures stratified by sputum eosinophil percent (23). A phase 2A RCT of dupilumab in eosinophilic asthma had only 15 patients who provided sputum samples (24), so no conclusions could be reached.

The anti-TSLP monoclonal antibody tezepelumab significantly reduces airway eosinophilia (25), and this may be the primary mechanism by which it reduces the rate of asthma exacerbations in moderate-severe asthmatics (26). Sputum eosinophilia may therefore be an excellent biomarker to predict patient response, but additional prospective studies are needed.

Sputum cytometry also facilitates the identification of paucigranulocytic and neutrophilic airway inflammation, which are perhaps subtypes of T2-low asthma (7). Though there are presently no biologic therapies available for patients with severe T2-low asthma, identification of these patients is crucial to prevent unnecessary therapeutic trials of anti-T2 biologics, which increases costs and delays further investigations and the institution of effective treatments (27, 28).

### Blood eosinophils

Blood eosinophils are easily obtained and thus have been the primary biomarker used in phase 3 trials of anti-IL5 biologics to identify severe eosinophilic asthma (21, 29–31). These trials have shown that generally patients with higher blood eosinophil counts have a greater response to IL5 signaling inhibition, probably because these patients are more likely to have significant airway eosinophilia. The threshold used for starting treatment with mepolizumab and benralizumab is ≥150 cells/μl and for reslizumab it is ≥400 cells/μl (32).

Despite the widespread use of blood eosinophils to select candidates for anti-IL5 therapy, there are key limitations of this approach. First, blood eosinophil counts were found to be poorly correlated with sputum eosinophils in patients on chronic systemic corticosteroids, leading to the underestimation of eosinophilia (and likely underuse of anti-IL5 therapy) in these patients (33). In the DREAM trial (in which ~1/3 of subjects were on maintenance systemic corticosteroids), a significant minority of patients showed discordance between blood eosinophils ≥150 cells/μl and sputum eosinophils >3% (34). Second, patients treated with anti-IL5 monoclonal antibodies who have suboptimal treatment responses may continue to have persistent airway eosinophilia, which is not reflected by blood eosinophil counts (18). Third, the reduction in blood eosinophils with IL5 therapy was also not found to correlate with ongoing treatment benefit (35). This discordance between blood and sputum eosinophilia in patients treated with chronic systemic steroids and anti-IL5 biologics is likely due to the suppression of systemic but not local airway eosinophilopoiesis (36), and, critically, suppression of local airway eosinophilopoiesis appears to be most important in determining the effectiveness of IL5 inhibition (37). For these reasons, we disagree with the European Respiratory Society/American Thoracic Society (ERS/ETS) severe asthma recommendations (3), which state that sputum eosinophils may not add additional value beyond blood eosinophils. Fourth, there is little evidence to support the blood eosinophil thresholds currently suggested for initiating anti-IL5 biologics, a problem which was also noted in the recent ERS/ATS recommendations (3).

Several studies have assessed whether blood eosinophil counts predict the response to omalizumab with conflicting results (38). For example, a large real-world retrospective study in patients from France found that there was no difference in patient response rate across all blood eosinophil levels from <150 cells/μl to >1,000 cells/μl (39). Multiple baseline characteristics of this cohort indicated that there was a strong indication for a biologic, so inappropriate patient selection does not appear to have been operative. In contrast, pooled analyses from large clinical trials in children and adults found a greater reduction in exacerbations in patients with ≥300 cells/μl compared to those with <300 cells/μl (40, 41). Exacerbations in autumn are an important source of morbidity for school-aged asthmatic children (42), and eosinophil count was correlated with benefit from seasonal as well as continuous omalizumab use (41).

Dupilumab had a greater effect on severe asthma exacerbations in moderate-severe asthmatics with blood eosinophils >300 cells/μl compared to 150–300 cells/μl and was no better than placebo in patients with <150 cells/μl (21). However, in oral corticosteroid dependent asthmatics, the prednisone sparing effect of dupilumab was not correlated with blood eosinophil count (23). Thus, low blood eosinophil counts in systemic corticosteroid-dependent patients do not reflect ongoing T2 airway inflammation amenable to dupilumab therapy and its use as a biomarker in these patients is not advisable.

Tezepelumab appears to have a greater effect on asthma exacerbation rates in patients with higher blood eosinophil counts (43), perhaps because its primary mechanism of action is to reduce airway eosinophilia (26). Blood eosinophils hence may be a useful biomarker to predict the efficacy of tezepelumab, but the same limitations affecting its predictive capacity for anti-IL5 drug efficacy would likely also be operative.

In summary, a substantial minority of patients who are likely to benefit from anti-IL5 therapy will not be identified using blood eosinophil counts, and blood eosinophil counts have little value in the assessment of patients who have persistent exacerbations despite anti-IL5 therapy. Higher blood eosinophil counts are associated with a greater reduction in exacerbations with tezepelumab and dupilumab (in patients not on systemic corticosteroids). There are conflicting data on the utility of blood eosinophil counts to predict response to omalizumab.

### Serum IgE

Omalizumab binds free IgE, preventing it from binding to IgE receptors on mast cells and basophils and downregulating FcεR1 receptors on basophils. The drug-IgE complexes are then removed by the reticuloendothelial system. Omalizumab was thus evaluated using a dosing schedule to achieve (1) a mean free IgE serum concentration of <25 ng/ml among all patients and (2) a free IgE <50 ng/ml in over 90% of patients, which is reflected in its approved dosing (44). However, robust data have demonstrated that baseline IgE does not predict the likelihood of response to omalizumab (45, 46). Serum IgE ≥30 IU/ml also does not predict the response to mepolizumab (47), tezepelumab (43) or dupilumab (48), and tezepelumab and dupilumab both reduce IgE, which further confounds the use of IgE if alternate therapies are being considered after a patient has failed these treatments. Total IgE is additionally influenced by the presence of common atopic comorbidities such as at atopic dermatitis and allergic rhinitis, and specific syndromes of which asthma is one component (e.g., allergic bronchopulmonary aspergillosis). Specific IgE to aeroallergens often fluctuate by season, making this measure difficult to interpret as well. For these reasons, serum IgE does not appear to be a useful biomarker for biologic selection in severe asthmatics.

### Fractional exhaled nitric oxide

Nitric oxide in the lung promotes dilation of the vasculature and airways and is considered a marker of airway inflammation. It is produced in the airway epithelium by the enzyme inducible nitric oxide synthase, which is in turn upregulated by IL13. The fraction of nitric oxide (FeNO) in exhaled breath can thus be used to assess patients for the presence of a T2 immune signature. FeNO is usually measured by chemiluminescence at the point of care. The patient exhales at a constant rate into an analyzer (at least 4 s for those <12 years and at least 6 s for those ≥12 years) and a value is provided in parts per billion (ppb). A FeNO >50 ppb in adults (or >35 ppb in children <12 years) suggests eosinophilic inflammation whereas a FeNO <25 ppb in adults (or <20 ppb in children <12 years) indicates absence of eosinophilic inflammation; the intermediate range 25–50 ppb in adults (or 20–35 ppb in children) is considered indeterminate and must be interpreted with caution (49). FeNO >50 ppb predicts glucocorticoid responsiveness in glucocorticoid naïve patients (50), but the utility of these thresholds in severe asthmatics being considered for biologic therapy has not been established. FeNO is also affected by several factors (e.g., tobacco smoke exposure, alcohol consumption, exercise, upper respiratory tract infections, and ingestion of nitrate rich foods) (49), which must be considered when interpreting the results.

Two phase 3 trials have demonstrated that FeNO predicts patient response to dupilumab (21, 23). In both the moderate-severe and corticosteroid-dependent asthma populations, higher FeNO was associated with a greater reduction in severe asthma exacerbations, and a threshold of 25 ppb appeared to differentiate responders from non-responders (51). In moderate-severe patients, FeNO>25 ppb also predict improvement in FEV1. However, FeNO did not predict the percentage reduction of oral glucocorticoids or the change in pre-bronchodilator FEV1in the systemic glucocorticoid dependent population (23).

In the phase 3 registration trial for tezepelumab, higher FeNO was associated with a greater reduction in asthma exacerbations, though importantly response was seen in both high- and low-FeNO subgroups defined using a threshold of 25 ppb (43). Tezepelumab also reduced FeNO, likely owing to its target TSLP being upstream of IL4 and IL13 in the allergic inflammation cascade.

FeNO generally correlates with airway eosinophilia, but the degree of correlation is insufficient for it to be useful in predicting the efficacy of anti-IL5 therapy. FeNO did not predict the response to mepolizumab in the DREAM trial (34), and this has been confirmed in a real-world cohort of patients treated with mepolizumab and benralizumab, even in those with FeNO >75 ppb (52). Thus, FeNO should be regarded as a parallel measure reflective of IL4 and IL13 activity, which is often but not invariably associated with IL5 signaling. Benralizumab reduces FeNO likely because it depletes eosinophils, which themselves produce IL13 (52), and this observation may be important if a patient has not responded adequately to benralizumab and is being assessed for dupilumab.

A recent observational study evaluated the correlation between FeNO suppression after high-intensity corticosteroid therapy, asthma control, and sputum eosinophilia (53). They reported that persistently elevated FeNO despite corticosteroid therapy was associated with persistent sputum eosinophilia and worse control. It is thus plausible that post-corticosteroid FeNO suppression rather than individual FeNO measurements may predict the efficacy of anti-IL5 therapy.

The utility of FeNO to predict the efficacy of omalizumab is unclear. The EXTRA trial, a large placebo controlled RCT (54), reported that patients with high FeNO (>19.5 ppb) had a significantly greater reduction in exacerbation rate with omalizumab compared to placebo (55). This finding, however, was driven by a lower exacerbation rate in the low-FeNO placebo group. The exacerbation rate between omalizumab treated patients with high and low FeNO was similar. A prospective observational study also found that outcomes in high and low FeNO patients (using 25 ppb as the threshold) treated with omalizumab were similar (56).

In conclusion, FeNO appears to be helpful in predicting patient response to dupilumab and tezepelumab but is not useful for predicting the responses to anti-IL5 therapies and its utility for anti-IgE therapy is questionable.

### Clinical characteristics

Clinical factors such as comorbidities are often considered when making biologic treatment decisions, either because the comorbid conditions may also respond to a specific asthma drug (e.g., dupilumab in a patient with concomitant atopic dermatitis) or because the presence of the comorbidity is thought to predict response to a particular asthma therapy. In the latter case, the clinical trial data have not convincingly shown comorbidities to be useful indicators for biologic therapy selection.

A meta-analysis of four different omalizumab studies showed that comorbidities did not affect efficacy of omalizumab (57). *Post hoc* analysis (GSK ID:209140) of data from the Phase IIb/III mepolizumab studies DREAM, MENSA, SIRIUS, and MUSCA showed reduced exacerbations and improved control irrespective of comorbid conditions, including upper respiratory conditions, psychopathologies, cardiovascular conditions, gastroesophageal reflux disease, diabetes mellitus, and obesity. Pooled analysis of the MENSA and MUSCA trial data further showed that severe eosinophilic asthma subgroups defined by age at asthma onset, lung function, airway reversibility and allergen sensitivities do not have different response to this biologic (58). Comorbid nasal polyposis has been shown to predict response to mepolizumab (59) and benralizumab (60). However, in a real-world effectiveness study of mepolizumab, comorbidities did not correlate with mepolizumab effectiveness (61).

The Severe Asthma Research Program (SARP), a prospective cohort study of patients with asthma (62), provides insight as to why such clinical factors are not easily incorporated into decision making. Using an unbiased classification algorithm, the cohort with severe disease was subdivided into three clusters based on clinical factors, including comorbidities. They found that specific sputum endotypes such as eosinophilic asthma did not correspond to a particular phenotypic cluster but rather were equally distributed among the three groups (63), indicating inflammatory subtyping using biomarkers is necessary.

Nonetheless, the use of clinical factors such as age of onset, prednisone-dependence, and comorbid nasal polyposis is a useful starting point (36, 63), particularly if sputum cytometry and FeNO are not available.

## Looking to the future

Biomarkers used today and specific phenotypes that may be associated with response to a specific agent have been described above. The utility of common laboratory biomarkers for predicting response to biologics is summarized in Table 1. Many of these standard biomarkers have been used to classify patients in the registration trials of the currently available biologics. It is interesting to keep in mind that with the current patient selection methods used for clinical trials, all currently approved biologics for severe asthma seem to have comparable efficacy in preventing severe exacerbations and improving other outcomes (64). Equally, these selection methods may not be optimal based on the mode of action of some biologics, which, for example, may have been the case for studies with an anti-IL23 antibody (65).

From the discussion so far, we have to conclude that induced sputum offers the best current biomarkers for response to anti-IL5 biologics, and possibly anti-TSLP, while we have less understanding of the biomarkers that predict response to the other currently available biologics. A recent international expert opinion paper attempted to give guidance on the approach to diagnosis and treatment for patients with severe asthma (32). These international experts acknowledge that “treatment algorithms in the current literature are complex and do not fully address the optimal treatment choice” leading them to conclude that “updated clinical treatment guidelines are needed for optimal, individualized management of this patient population.”

If we accept the premise that we have yet to realize the vision of precision medicine in severe asthma, we then consider how to further this goal. Two approaches, we believe, can be very helpful in this quest. First, we should look into published secondary analyses of large studies on the treatment of severe asthma and identify potential biomarkers that could be tested in studies designed specifically for this purpose. Second, we should look into identifying novel biomarkers through specifically designed studies that may generate new approaches to phenotyping asthma patients and may identify new and/or more specific endotypes. This new knowledge should then be tested directly in studies to validate the utility of these biomarkers to predict response to therapy. Here, we will discuss prospective biomarkers that may further the goal of precision therapy in severe asthma in the future (Table 2).

**Table 2:** Summary of potential novel biomarkers in asthma.

**Biomarker**	**Potential Utility**
Mast cell mediators (e.g., tryptase)	Predict response to TKIs or omalizumab
IL13-inducible gene products or IL13 gene signature	Predict response to anti-IL13 biologics
Th17 effectors (e.g., IL17A) and stimulators (e.g., IL23)	Identification of T2-low asthma Predict response to anti-IL23 biologics
INFγ and IL6 gene signatures	Novel disease endotypes
Airway concentrations of biologics	Assess adequacy of biologic dosing

INFγ, interferon gamma; TKI, tyrosine kinase inhibitor.

### Mast cell driven asthma: Are there specific biomarkers for a mast cell asthma endotype?

Mast cells (MC) are found throughout the lung including within the epithelium, submucosa and the smooth muscle layers in the airways and their characteristics, location and activation status may be altered in severe asthma (66). MC activation through FcεRI engagement and cross linking by IgE and antigen is a key mechanism of allergic asthma through the release of a large array of mast cell mediators that can bronchoconstriction by triggering smooth muscle cells, but also affect many other lung and airway cells. However, MC can also be involved in the pathogenesis of non-atopic asthma following activation by IgE-independent triggers. For example, a U-BIOPRED study has shown that transcriptional signature of IL33 activated MC in bronchial biopsies is associated with neutrophilic asthma, in contrast to FcεRI-activated MC signature that is associated with eosinophilic asthma (67). Another study has identified 8 mast cell-related genes in induced sputum of patients with asthma that correlated with eosinophilic disease, lung function or FeNO (68). These observations indicate that there may be more than one MC-dependent asthma endotypes, and we know very little weather these endotypes have differential response to the current biologics used for asthma therapies. Omalizumab, for example, targets free IgE and therefore prevents FcεRI-mediated mast cell and basophil activation and may prevent the effects of mast cell activation in severe asthma. Would MC signatures and expression of MC-specific genes mark a mast cell-dependent endotype of T2 asthma that may respond better to anti-IgE therapies? Only testing these signatures in prospective studies will tell us if this is the case.

MC-dependent asthma endotypes may also respond to agents that target MC activation. Tyrosine kinase inhibitors (TKIs) are such agents that can prevent mast cell growth and activation through c-kit, and possibly other MC tyrosine kinases, inhibition and under certain circumstances can decrease mast cell burden (69, 70). There is limited information on the efficacy of TKIs in severe asthma. One study enrolled patients with severe asthma that had poorly controlled disease and persistent airway hyperresponsiveness despite treatment with high dose inhaled corticosteroids and one more controller medication, and treated these patients with imatinib for 24 weeks (71). The intervention decreased AHR, serum tryptase, as a marker of mast cell activation, and airway mast cell numbers. Patients on imatinib also had numerically fewer asthma exacerbations, higher morning and evening peak expiratory flows, and greater improvements in patient-reported outcomes than patients on placebo, but these differences were not statistically significant. A recent report in a form of an abstract (72), suggests that masitinib, a TKI that targets c-kit and other tyrosine kinases important in MC functions, decreased the rate of asthma exacerbations in patients with severe asthma that is not controlled with the use of oral corticosteroids. These studies did not select patients for a MC-dependent phenotype or endotype. An ability to select people more likely to respond to anti-MC therapies may improve the efficacy of these agents.

Since MC play a role in asthma pathogenesis, MC mediators in serum may be useful biomarkers. MC tryptase is a tetrameric trypsin-like protease released exclusively by MC (73). Four tryptase isoforms exist in humans and there are two isoforms, α- and β-tryptase, that are released by MC. Among them, β-tryptase is for the most part enzymatically active, while α-tryptase is inactive. Individuals have a variable number of active tryptase genes (73). MC tryptase has been implicated in the pathophysiology of asthma (74). While BAL tryptase is elevated in both patients with moderate and severe asthma, serum tryptase is elevated (compared to normal controls) only in patients with severe asthma (75). In addition, both BAL and plasma tryptase elevation in severe asthma are independent of other classic T2 markers, such as blood eosinophils. Tryptase may have an autocrine effect on mast cell degranulation, so elevated active tryptase may activate mast cells independent of FcεRI crosslinking and therefore induce mast cell degranulation that will not be prevented by omalizumab. A recent study showed that omalizumab was effective only for subjects with severe asthma with a low number of active tryptase alleles and had no effect in those with higher allele numbers (75). Perhaps then genotyping for active tryptase alleles would prove to be a useful biomarker to improve patient selection for omalizumab.

### Can IL13-inducible genes be used as a marker of response to anti-IL13 agents?

Periostin has been suggested as a biomarker for response to lebrikizumab already from early studies on the efficacy of lebrikizumab in asthma (76), although it has not been validated in all subsequent studies (77). Other studies showed that a SNP in the IL13 gene (+2044G>A that translates into IL13Q144 protein) of variable frequency in various populations (78) may affect affinity of anti-IL13 monoclonal antibodies (both lebrikizumab and tralokinumab) with IL13 and may therefore affect the efficacy of the antibodies in treating severe asthma (79). Levels of other IL13-inducible genes (e.g., dipeptidyl peptidase 4), or multigene signatures downstream of the IL13 pathway may be an even better biomarker for response to anti-IL13 biologics. It is possible similarly that IL5 or IL4 response signatures in serum (ideally due to ease of sampling) or in respiratory secretions (with potentially increased specificity, but more difficult to access) may be good markers for response to biologics affecting those pathways.

### Are there biomarkers for the response to tezepelumab?

A recent *post hoc* analysis of biomarkers from the PATHWAY phase IIb study of tezepelumab was interesting in that none of the established T2 biomarkers seem to be associated with response to treatment (80). IL5, IL13 and FeNO levels as well as blood eosinophil counts responded to treatment with tezepelumab within 4 weeks, but increased levels of these biomarkers at the beginning of the study did not correlate with drug efficacy. In fact, separating the subjects into those with levels above or below the median for blood eosinophil count, FeNO, serum IgE, IL5, IL13, periostin, TARC, and TSLP showed that in each case the two groups had similar response to the drug. Therefore, new biomarkers may be needed to help us identify patients that will respond to tezepelumab. This may become clearer as this new biologic is incorporated into our therapeutic options.

### Are there biomarkers for non-T2 asthma?

IL23 promotes the development of Th17 lymphocytes, and these cells elaborate IL17A, which is important for the recruitment of neutrophils to the airways in non-T2 asthma (81). However, a recent phase 2A RCT of risankizumab, an anti-IL23 monoclonal antibody, showed no effect on asthma exacerbations (65). Subgroup analysis did not show benefit in those with neutrophilic airway inflammation, but the trial was not powered for this purpose. Transcriptomic analysis in this study showed that risankizumab down regulated the activation of Th1 and Th17 cells as well as NK and CD8 cells, indicating that there may be a subgroup of patients that could benefit from this biologic if these cells were important for the development of disease in these patients. Will a follow up study try to identify the subgroups of patients that may benefit from this biologic, or will these data spur the termination of the program? These are interesting questions that may be important for many new therapeutic options and that need to be answered soon.

### Can new biomarkers be identified from asthma -omic studies

Omics studies have generated a wealth of information on asthma pathogenesis and on disease endotyping over the last few years (82). The various -omics studies in most case use data generated from the analysis of samples recovered from the target tissues during bronchoscopy, such as epithelial and BAL cells or biopsies. However, information from these studies is quite complex and even though -omics analyses are becoming easier to access for clinical purposes, use of such assays for clinical characterization of patients with severe asthma will not be available in the foreseeable future for the vast majority of patients, due to the difficulty, cost and risks of performing bronchoscopies.

Airway epithelial cells and BAL cells are easily accessible through bronchoscopy and a number of studies from European (U-BIOPRED) and North American (SARP) cohorts have identified through transcriptomic approaches characteristics of airway epithelium that may associate with specific disease phenotypes and endotypes. A microarray study identified an IL6 high phenotype of asthma by the presence of IL6 inducible genes in microarrays of bronchial brushings (83). This group of patients with severe asthma is characterized by frequent exacerbations, peripheral and airway eosinophilia, increased immune cell infiltration of the airway submucosa and evidence of defective barrier function, but does not correlate with the group with T2 high epithelial signature that has been previously defined (84). Reanalysis of a previous cohort from a study that evaluated protein levels in induced sputum of patients with severe asthma (85), identified a similar group of subjects with increased levels of IL6-induced proteins in induced sputum. Microarray data from epithelial cell brushings form subjects participating in the SARP program analyzed through machine learning approaches identified four participant clusters that separated healthy controls from asthmatics and having a cluster that included most of the subjects with severe asthma (86). These clusters would be indistinguishable if traditional T2 biomarkers were used for grouping the subjects.

Microarray data of cells from BAL of patients with asthma also identifies a IFNγ high subgroup of asthmatics (87). This phenotype was enriched in subjects with severe disease and had decreased lung function. The two groups however, had no differences in sputum and blood eosinophil counts, total serum IgE or FeNO levels indicating that an IFNγ phenotype may spread across various levels of other biomarkers we use to characterize our patients as T2 high and may complicate the selection of patients for T2 targeted therapies. Another study analyzed gene expression patterns in BAL and airway epithelia cells and found a number of genes associated with asthma severity (88). An interesting observation was that the expression pattern was affected by usual asthma medication, such as b-agonists. Another caveat of such studies could be the fact that transcription associated patterns may not be stable over time even in patients that are free of exacerbations and other acute disease changes (89).

Studies like the U-BIOPRED and consortia like SARP have given a wealth of information on -omics in asthma. The big question now is how can we use these studies to improve our ability to choose the right medication for the right patient. The hope is that these studies will define endotypes and these endotypes will inform biologic selection for each individual patient using knowledge of disease mechanisms. The question however remains whether we are at the point where this expectation can become reality. And if we are far from this time in the natural history of our understanding of asthma, should we change our focus and the studies we do. If this is the case, then who should be responsible to follow these new pathways? Should national funding bodies change their focus, or should we “mandate” industry to perform some of these studies?

### Can airway drug concentrations be used as biomarkers?

Given the importance of local airway eosinophilopoiesis in severe asthma (37), local concentrations in the airways may be particularly important for optimal effect of anti-IL5 biologics. However, there are no published pharmacokinetic studies of asthma biologics that measured drug concentrations in the airways in addition to the blood. Indeed, there is evidence that inadequate dosing of mepolizumab may be the reason for therapeutic failure in some patients (90). This, we believe, is an important gap in the literature. Assays for the measurement of drug concentrations should be made available by industry to allow researchers to further test this hypothesis. If airway drug concentrations are well correlated with therapeutic benefit, such measurements could potentially be adapted to clinical practice using induced sputum samples.

### How will we identify potential future biomarkers?

We do not see this article as a detailed discussion of all the biomarkers that show promise for severe asthma, something that has been done by others recently (91–93), and we have not done that in our discussion so far. Our point is more to try to identify approaches that could allow better use of the current available therapies as our controversial for many of our readers title says.

The unanswered question is how can we go forward and perform the studies to increase our ability to use the current medications. We can understand the industry may not have an incentive to identify further biomarkers for therapies that are already approved for the general population of T2 severe asthma and though this approach decreases their target population.

## Conclusion

Severe asthma is a heterogenous airways disease. Recent insights into the pathophysiology of airway inflammation in asthma has led to the development of multiple biologicals targeting specific cytokines, and these have significantly improved outcomes for many severe patients. Sputum eosinophils appear to be the most informative biomarker for predicting the efficacy of biologicals targeting the IL5 pathway. Blood eosinophils can be a useful surrogate but a significant minority of patients who would benefit are likely to be missed, particularly if they are on systemic corticosteroids, and it is these severe patients who would derive the greatest benefit from anti-IL5 therapy. Sputum cytometry is also useful when assessing why a patient has failed to respond adequately to an anti-IL5 drug and is likely to be helpful for selecting patients for tezepelumab, but further data are needed. FeNO appears to be the most useful biomarker to identify dupilumab responders and blood eosinophils may also be helpful in patients not using systemic corticosteroids. Other than the need for systemic corticosteroids (which indicates an anti-IL5 drug should be used), there are no good clinical or biochemical indicators to inform the choice between omalizumab and anti-IL5 therapies in patients who qualify for both drugs. Novel biomarkers such as airway mast cell tryptase, IL13 inducible genes, and others show some promise to aid in biologic selection. Without the discovery and validation of new biomarkers, the goal of precision medicine in severe asthma will remain elusive.

## Author contributions

All authors conceptualized, drafted, and approved the final manuscript. All authors contributed to the article and approved the submitted version.

## Conflict of interest

Author AA was supported by the Canadian Institutes of Health Research/Canadian Allergy, Asthma, and Immunology Foundation/AstraZeneca/Allergen NCE Emerging Scientist Award in Allergic Asthma. The remaining author declares that the research was conducted in the absence of any commercial or financial relationships that could be construed as a potential conflict of interest.

## Publisher's note

All claims expressed in this article are solely those of the authors and do not necessarily represent those of their affiliated organizations, or those of the publisher, the editors and the reviewers. Any product that may be evaluated in this article, or claim that may be made by its manufacturer, is not guaranteed or endorsed by the publisher.
